# Imaging Non-Specific Wrist Pain: Interobserver Agreement and Diagnostic Accuracy of SPECT/CT, MRI, CT, Bone Scan and Plain Radiographs

**DOI:** 10.1371/journal.pone.0085359

**Published:** 2013-12-30

**Authors:** Martin W. Huellner, Alexander Bürkert, Klaus Strobel, María del Sol Pérez Lago, Lennart Werner, Urs Hug, Urs von Wartburg, Burkhardt Seifert, Patrick Veit-Haibach

**Affiliations:** 1 Department of Radiology and Nuclear Medicine, Lucerne Cantonal Hospital, Lucerne, Switzerland; 2 Department of Hand and Plastic Surgery, Lucerne Cantonal Hospital, Lucerne, Switzerland; 3 Division of Biostatistics, Institute for Social and Preventive Medicine, University of Zurich, Zurich, Switzerland; University of Otago, New Zealand

## Abstract

**Purpose:**

Chronic hand and wrist pain is a common clinical issue for orthopaedic surgeons and rheumatologists. The purpose of this study was 1. To analyze the interobserver agreement of SPECT/CT, MRI, CT, bone scan and plain radiographs in patients with non-specific pain of the hand and wrist, and 2. to assess the diagnostic accuracy of these imaging methods in this selected patient population.

**Materials and Methods:**

Thirty-two consecutive patients with non-specific pain of the hand or wrist were evaluated retrospectively. All patients had been imaged by plain radiographs, planar early-phase imaging (bone scan), late-phase imaging (SPECT/CT including bone scan and CT), and MRI. Two experienced and two inexperienced readers analyzed the images with a standardized read-out protocol. Reading criteria were lesion detection and localisation, type and etiology of the underlying pathology. Diagnostic accuracy and interobserver agreement were determined for all readers and imaging modalities.

**Results:**

The most accurate modality for experienced readers was SPECT/CT (accuracy 77%), followed by MRI (56%). The best performing, though little accurate modality for inexperienced readers was also SPECT/CT (44%), followed by MRI and bone scan (38% each). The interobserver agreement of experienced readers was generally high in SPECT/CT concerning lesion detection (kappa 0.93, MRI 0.72), localisation (kappa 0.91, MRI 0.75) and etiology (kappa 0.85, MRI 0.74), while MRI yielded better results on typification of lesions (kappa 0.75, SPECT/CT 0.69). There was poor agreement between experienced and inexperienced readers in SPECT/CT and MRI.

**Conclusions:**

SPECT/CT proved to be the most helpful imaging modality in patients with non-specific wrist pain. The method was found reliable, providing high interobserver agreement, being outperformed by MRI only concerning the typification of lesions. We believe it is beneficial to integrate SPECT/CT into the diagnostic imaging algorithm of chronic wrist pain.

## Introduction

Chronic hand and wrist pain is a common clinical issue for hand surgeons, orthopaedic surgeons and rheumatologists. Physicians are often challenged by symptoms that are hard to allocate precisely and may also change in the course of time. After the clinical examination, the imaging work-up usually starts with plain radiographs. Cross-sectional modalities such as MRI (Magnetic resonance imaging) are frequently performed subsequently. MRI has the major advantage of showing early damage to intra-articular and extra-articular soft tissue such as cartilage, ligaments and tendons, which is a common reason for wrist pain. Therefore, MRI is recommended by expert consensus opinion if radiographs are negative [1]. However, some patients are still lacking an appropriate diagnosis after MRI, and hence, adequate therapy. Identifying the responsible pathology is especially difficult in patients having clinically non-specific wrist pain and multiple lesions. 

Hybrid SPECT/CT (Single photon emission computed tomography / computed tomography), which has emerged in the last years, provides information both about the morphological structure and the metabolic activity of lesions, and allows for an exact anatomical localisation of pathological lesions [2,3]. 

The accuracy of observers and the interobserver agreement in MRI depends on the type of lesion in the hand and wrist, and may vary even in experienced radiologists [4,5]. In a recent pilot study, it was demonstrated that SPECT/CT is more specific than MRI concerning the detection of clinically relevant lesions in patients with non-specific wrist pain [6]. It was also shown that SPECT/CT has a higher interobserver and intraobserver reliability than CT, bone scan, or a combination of both in patients with non-specific pain of the foot and ankle [7]. To date, the experience with SPECT/CT of the hand and wrist is limited, and there are no major studies focusing on the interobserver agreement of SPECT/CT compared with other established imaging modalities. 

Thus, the purpose if this study was 1. to analyze the interobserver agreement of SPECT/CT, MRI, CT, bone scan and plain radiographs in patients with non-specific pain of the hand and wrist, and 2. to assess the diagnostic accuracy of these imaging methods in this selected patient population. 

## Materials and Methods

### Patients

Thirty-two consecutive patients (median age: 38 years, range 18 to 73 years, 19 females, 13 males) with non-specific pain of the hand or wrist were included. Ethical approval was waived by the approving IRB (Cantonal Ethics Committee) due to the retrospective nature of the study. For the same reason, written consent was not obtained from subjects and waived by the approving IRB. The diagnosis of non-specific wrist pain was made by the referring hand surgeon, based on patient history, clinical examination, plain radiographs and clinical guidelines [8]. Conservative therapy failed to improve the symptoms in all patients. Continuance of clinical symptoms was present in all patients at the time of all imaging procedures.

Prior trauma had occurred in 15 patients (47%; 7 females, 8 males; median: 351 / 379 / 446 days before plain radiography / MRI / SPECT/CT). The first onset of clinical symptoms was nine months before plain radiographs (median: 259 days), MRI (218 days), and SPECT/CT (254 days). Most patients (78%; 25/32) suffered from pain in the dominant hand. Nineteen patients (59%; 19/32) perceived symptoms only during exercise, 13 patients also at rest. The location of symptoms was ulnar-sided or ulnovolar (15 patients), radial-sided (11), dorsally (5) and generally at the wrist (1). All diagnostic examinations were performed within 4 months. The median time interval between plain radiographs and MRI / SPECT/CT was 32 / 34 days, and between MRI and SPECT/CT 37 days. All patients underwent plain radiography, planar early (bone scan) and late-phase imaging (SPECT/CT including bone scan and CT), and MRI. 

### Imaging procedures

Plain radiographs of the wrist consisted of one anteroposterior and one lateral image. All MRI examinations were performed on a 3 Tesla scanner (Achieva 3.0T, Philips Healthcare, Best, The Netherlands). Depending on the clinical question, patients were examined by indirect MR-arthrography, or direct two-compartment MR-arthrography under fluoroscopic surveillance (direct MR-arthrography: Artirem, Guerbet, Villepinte, France, and Iopamiro 300, Bracco, Milano, Italy; mixing ratio 7:3; indirect MR-arthrography: 7.5 ml Gadovist, Bayer Healthcare, Leverkusen, Germany, followed by saline flush). Our standard MRI protocol included water selective GRE (Gradient-recalled echo) sequences (S3D WATS (water selective excitation), TR/TE (time to repetition / echo) 19/92ms, section thickness 1mm, FOV (field of view) 100mm, matrix 224x224mm), coronal (TR/TE 3154/30ms, section thickness 1.5mm, FOV 126mm, matrix 480x480mm) and sagittal PDw (proton density-weighted) sequences (TR/TE 3803/30ms, section thickness 1.5mm, FOV 126mm, matrix 320x320mm) with and without fat suppression (SPIR ,spectral presaturation with inversion recovery), TR/TE 2778/30ms), and axial PD aTSE (turbo spin echo) (TR/TE 5914/30ms, section thickness 2mm, FOV 100mm, matrix 352x352mm). 

SPECT/CT imaging was performed on a hybrid SPECT/CT system with a built-in flat-panel CT component (BrightView XCT, Philips Healthcare) after injection of a mean activity of 650 MBq ^99m^Tc-DPD (Technetium-99^m^-3,3-diphosphono-1,2-propanedicarboxylic acid, Teceos, Behringwerke, Marburg, Germany). Early-phase planar images were acquired directly after injection during 5 minutes (matrix 256x256mm, FOV 40cm). Late-phase images (matrix 256x256mm, FOV 40cm), SPECT (matrix 512x512mm, FOV 40cm) and CT images (matrix 512x512mm, FOV 40cm, 120 kV, 85 mAs, automated dose modulation, 0.5s rotation time, slice thickness 0.33mm) were acquired after 3 hours. SPECT and CT images were reconstructed by iterative reconstruction, CT images in all three planes as 1 mm slices. SPECT and CT images were fused by an automated software algorithm on a dedicated workstation (Extended Brilliance Workspace, Philips Healthcare). Both planar bone scans and CT for read-out were derived from the examination performed on the SPECT/CT machine. 

### Image evaluation

A region-based evaluation of all five modalities was carried out by four radiologists and / or nuclear medicine physicians using the local PACS (Merlin PACS, Phönix-PACS, Freiburg, Germany). Reader 1 (L.W.) had one year of experience in radiology, including 4 months of CT. Reader 2 (M.P.) had four years of experience in radiology, thereof two years in CT, and nine years in nuclear medicine. Reader 3 (K.S.) had 12 years of experience in radiology and 15 years in nuclear medicine. Reader 4 (A.B.) had nine years of experience in radiology. All images were analyzed independently, and in a blinded and randomized fashion. The readers were provided with a brief clinical history, including information about prior trauma, the time of onset and the location of symptoms, and if the dominant or the non-dominant wrist was affected during exercise or also at rest. Readers were advised to focus on the relevant lesion, i.e. the lesion being responsible for the patient's symptoms and requiring treatment or a change of treatment. 

Several categories of pathology were assessed:

• detection of the clinically relevant lesion(s) (yes / no ),
• its exact location or joint site (designation of the respective location on a standardized read-out form with 74 possible sites per hand / wrist),
• the predominant type (bone, cartilage, synovia, tendon, ligaments, capsule, or any combination), and
• the assumed etiology of pathology (posttraumatic, degenerative, primary constitutional, inflammatory, stress, vascular, or any combination).

The standard of reference consisted of complete clinical examination and all imaging procedures performed, with a mean clinical follow-up period of 20 months in 21 patients. During this period of time, every patient had at least one further clinical examination by a hand surgeon. The remaining eleven patients additionally underwent arthroscopy during their mean clinical follow-up period of 16 months. If complete clinical and diagnostic work-up failed to detect the cause of the pain in a patient, the standard of reference was rated as negative. Hence, if the readers rated an imaging method as negative in this patient, the result was defined as true negative. Results were expressed as mean detection rate, which means that a lesion detected correctly by e. g. one of two readers was rated as 50% mean detection rate.

### Statistical analysis

The interobserver agreement was determined by calculating the kappa for denominating the relevant lesion, and location, type and etiology of pathology (see evaluation above). Results of each diagnostic method were subdivided according to the level of training of the readers (readers with expert / basic knowledge in radiology: 3, 4 / 1, 2; expert / basic knowledge in nuclear medicine: 2, 3 / 1, 4). Kappa for all analyzed imaging criteria are presented as kappa for two readers (agreement between two particular readers) or as mean of the four kappa for each experienced versus each inexperienced reader (agreement between experienced and inexperienced readers). Kappa values including 95% bias corrected bootstrap confidence intervals (CI) were computed using the procedure kapci in Stata 11.2 (StataCorp, College Station, Texas, USA). Moderate agreement was defined if between 0.41-0.60, substantial agreement if between 0.61-0.80, and almost perfect agreement if >0.80) [9]. 

The diagnostic accuracy and the area under the receiver operating characteristics (ROC) curve were calculated for detecting the relevant lesion according to the standard of reference mentioned above. A score was collected for experienced and inexperienced readers concerning lesion detection and localization, broken down to lesion type and geographic distribution, respectively. Differences between experienced and inexperienced readers in lesion detection were analyzed by Wilcoxon signed ranks test, based on a mean detection rate calculated for each reader group. A p-value of <0.05 was considered statistically significant. 

## Results

### Clinical follow-up

Twenty-seven of the 32 patients had a lesion that was responsible for their symptoms, whereas in 5 patients no causative lesion could be found. An overview of all lesions and mean detection rates is given in table **1**. 

**Table 1:** Relevant lesions according to the standard of reference; mean detection rate by all modalities for experienced and inexperienced readers.

**Final diagnosis**	**Number of relevant lesions / standard of reference**	**Percentage of relevant lesions detected (mean percent values based on two readers in each group)**									
		**Plain radiographs**		**Bone scan**		**CT**		**SPECT/CT**		**MRI**	
		Experienced readers	Inexperienced readers	Experienced readers	Inexperienced readers	Experienced readers	Inexperienced readers	Experienced readers	Inexperienced readers	Experienced readers	Inexperienced readers
Ulnocarpal impaction	3	33	33	33	17	66	33	66	33	83	50
Ulnocarpal impaction with DRUJ osteoarthritis	1	100	100	50	50	50	50	100	100	100	50
DRUJ chondromalacia	1	0	0	0	0	0	0	0	0	0	0
DRUJ instability	1	50	100	0	0	100	100	100	0	100	100
Scapholunar instability	1	50	0	0	0	0	50	0	0	50	0
TFCC tear	1	0	0	0	0	0	0	100	0	100	50
Radiocarpal osteoarthritis	1	100	100	100	100	100	100	0	0	50	50
Pisotriquetral osteoarthritis	1	50	0	0	0	0	0	0	0	0	0
Osteoarthritis of STT joint	1	0	0	0	0	0	0	100	0	0	0
Osteoarthritis of 1^st^ CMC joint	3	66	66	83	66	66	50	100	66	66	17
Type II lunate bone with chondromalacia	1	0	0	0	50	0	0	100	0	100	100
Carpal boss	1	100	0	50	50	100	0	100	50	100	50
Radiocarpal dorsal ganglion cyst	2	0	0	0	0	0	0	0	0	100	25
Intraarticular radius fracture	1	50	50	50	50	100	100	100	100	100	50
Fracture of the hamate hook	1	0	0	100	50	0	0	100	100	100	0
Posttraumatic bone remodeling (radius, ulna)	2	0	0	75	0	0	0	100	75	0	0
Stress reaction	1	0	0	0	0	0	0	100	0	0	0
Osteomalacia of the lunate bone	2	0	0	50	50	50	0	100	100	100	50
CRPS	1	0	0	100	50	0	0	100	0	0	0
ECU tendinitis	1	0	0	0	0	0	0	0	0	100	0
		**Mean detection rate ± SD**									
*All lesions*	*27 (100%)*	*30% ± 5%*	*24% ± 3%*	*39% ± 13%*	*28% ± 13%*	*35% ± 3%*	*24% ± 8%*	*74% ± 0%*	*41% ± 5%*	*65% ± 3%*	*30% ± 26%*
*No pathology*	*5 (100%)*	*20% ± 0%*	*60% ± 0%*	*70% ± 14%*	*90% ± 14%*	*50% ± 14%*	*60% ± 0%*	*90% ± 14%*	*60% ± 57%*	*10% ± 14%*	*80% ± 28%*
**Combined**	**32 (100%)**	**28% ± 4%**	**30% ± 2%**	**44% ± 13%**	**38% ± 9%**	**38% ± 4%**	**30% ± 7%**	**77% ± 2%**	**44% ± 4%**	**56% ± 4%**	**38% ± 18%**
		**p = 0.79**		**p = 0.29**		**p = 0.16**		**p < 0.001**		**p = 0.06**	

Results are given as mean per-cent values. TFCC: triangular fibrocartilage complex, DRUJ: distal radioulnar joint, STT: scaphotrapeziotrapezoid, ECU: extensor carpi ulnaris, CMC: carpometacarpal, CRPS: chronic regional pain syndrome, SD: standard deviation.

### Diagnostic accuracy

The diagnostic accuracy of plain radiographs was insufficient in all readers (range 25% - 31%). Bone scans also showed no valuable performance, with the highest diagnostic accuracy achieved being 53% (experienced reader). In CT, the highest diagnostic accuracy was 41% (experienced reader). In contrast, SPECT/CT performed well yielding diagnostic accuracies of 75% to 78% for experienced readers, and still 41% to 47% for inexperienced readers. However, MRI yielded only insufficient diagnostic accuracy (experienced readers: range 53% - 59%, inexperienced: 25% - 50%). SPECT/CT was significantly more accurate for experienced than for inexperienced readers (p < 0.001), while there was borderline significance concerning MRI (p = 0.06). The most specific modality for the evaluation of patients with clinically non-specific wrist pain by experienced readers was SPECT/CT (mean specificity 90%), whereas MRI yielded only poor specificity (10%). Both modalities were the only ones with reasonable sensitivity (SPECT/CT 74%, MRI 65%). Detailed results are listed in table **2**. 

**Table 2:** Binary classification of lesion detection by all modalities for experienced and inexperienced readers.

	**Plain radiographs**		**Bone scan**		**CT**		**SPECT/CT**		**MRI**	
	Experienced readers	Inexperienced readers	Experienced readers	Inexperienced readers	Experienced readers	Inexperienced readers	Experienced readers	Inexperienced readers	Experienced readers	Inexperienced readers
**Sensitivity**	30%	24%	39%	28%	35%	24%	74%	41%	65%	30%
**Specificity**	20%	60%	70%	90%	50%	60%	90%	60%	10%	80%
**Accuracy**	28%	30%	44%	38%	38%	30%	77%	44%	56%	38%
**Positive predictive value**	66%	76%	86%	95%	79%	76%	98%	88%	80%	93%
**Negative predictive value**	5%	13%	18%	19%	13%	13%	39%	14%	05%	17%
**Area under the curve (95% CI)**	0.25 (0.09 - 0.41)	0.42 (0.22 - 0.62)	0.54 (0.35 - 0.74	0.59 (0.41 - 0.77)	0.43 (0.23 - 0.62)	0.42 (0.22 - 0.62)	0.82 (0.69 - 0.95)	0.50 (0.31 - 0.70)	0.37 (0.20 - 0.54)	0.55 (0.36 - 0.74)

Results are given as per-cent values for each modality, averaged over the respective two readers per reader group.

**Figure 1:**
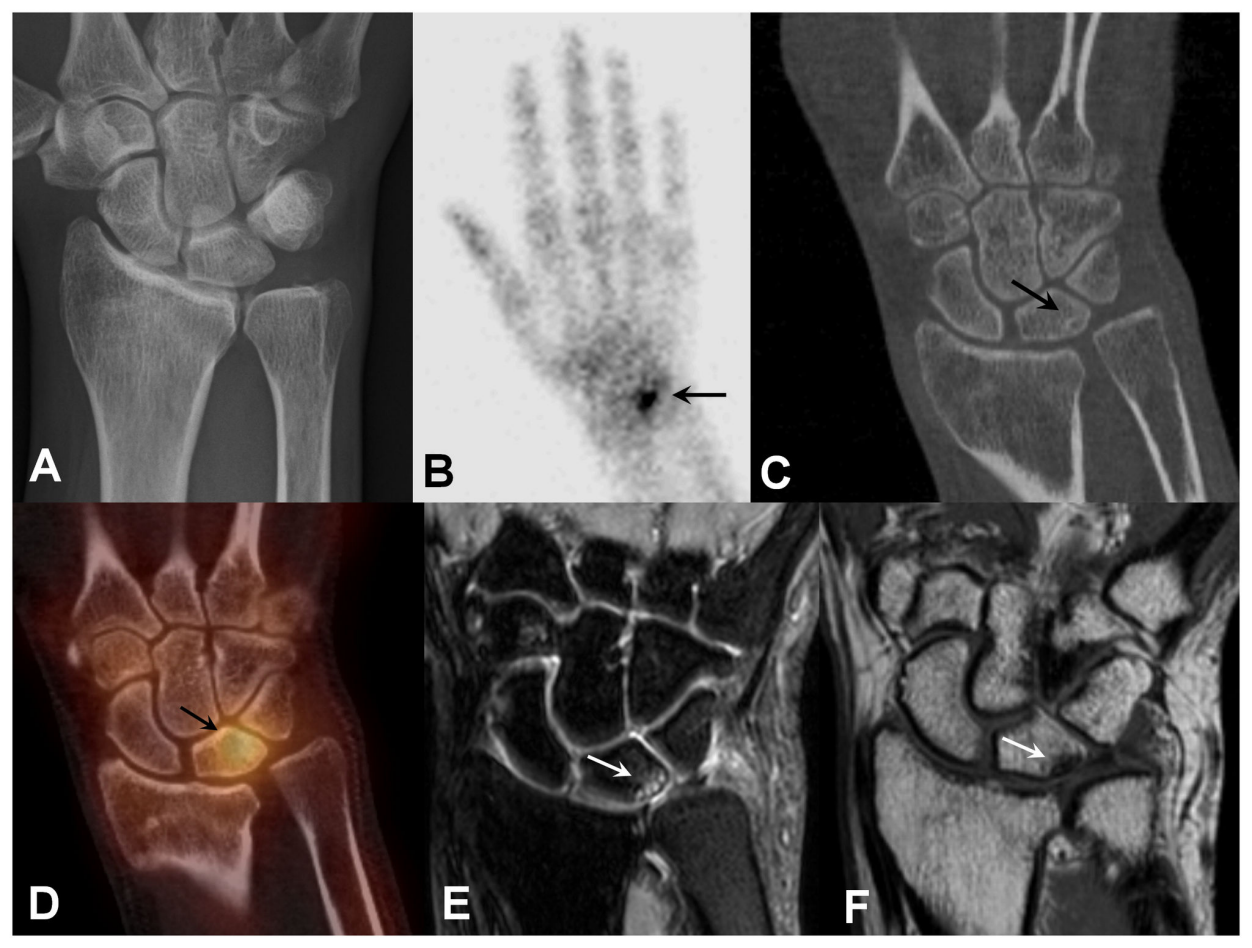
50-year-old female with ulnocarpal impaction syndrome. Relative overlength of the distal articular surface of the ulna on plain radiograph with localized geographic cystic-sclerotic changes in the medial proximal pole of the lunate and overprojection of TFCC calcification (a), focally increased radiotracer uptake in the carpal region on bone scan (b, arrow), subcortical cyst with rim sclerosis at the proximal ulnar pol of the lunate bone on CT (c, arrow), subcortical cyst displaying tracer accumulation on SPECT/CT fusion image (d, arrow), several subchondral cysts with adjacent alterations of bone marrow signal in the proximal ulnar pol of the lunate bone in PDw SPIR (e, arrow) and T1w image (f, arrow) on MRI.

**Figure 2:**
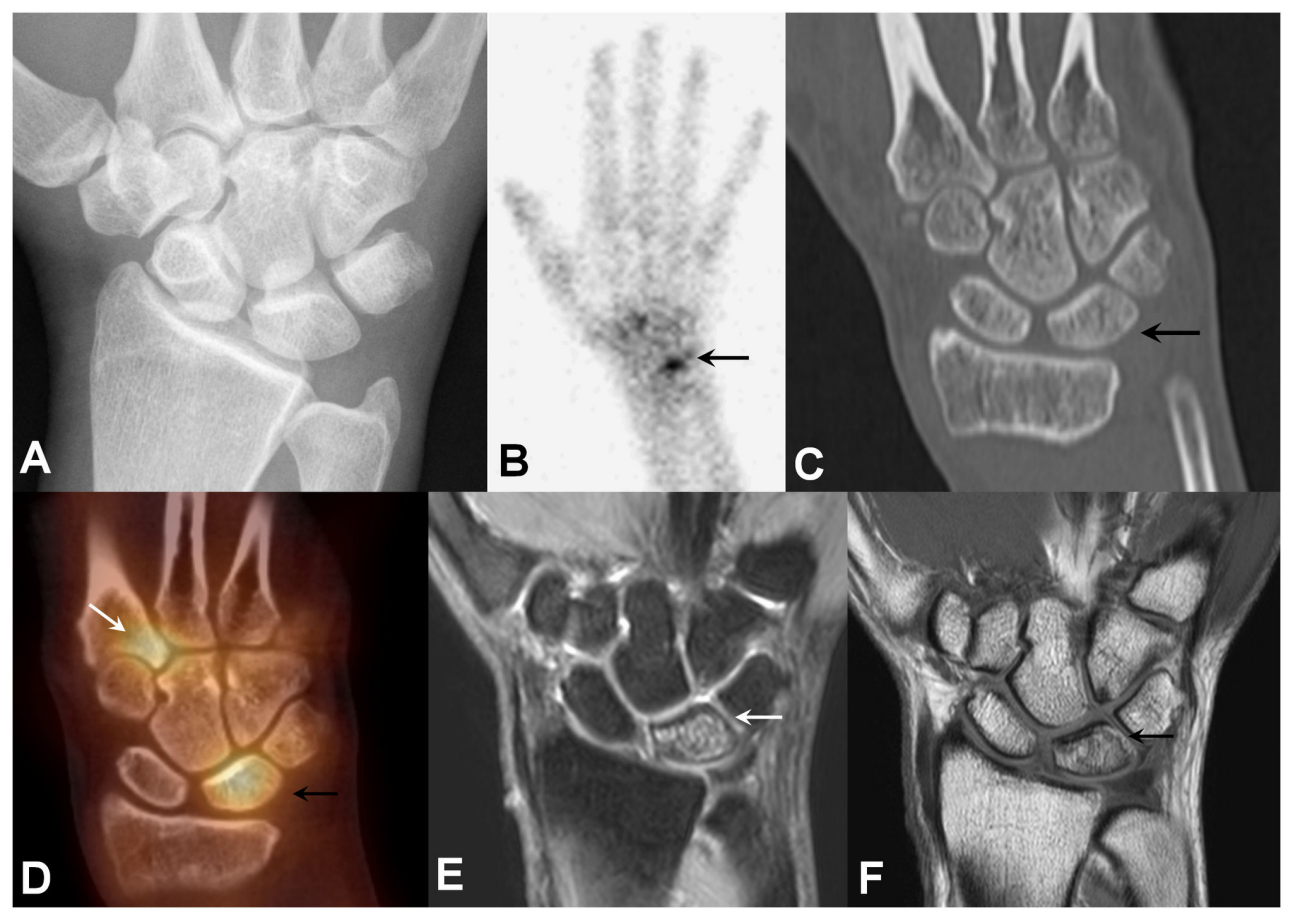
44-year-old female with osteomalacia of the lunate bone. Relative shortness of the distal articular surface of the ulna on plain radiograph (a), focally increased radiotracer uptake in the carpal region on bone scan (b), slightly hyperdense and coarse trabecular structure of the lunate bone on CT (c), tracer accumulation throughout the lunate bone on SPECT/CT fusion image (d), altered signal of lunate bone marrow in PDw SPIR (e) and T1w image (f) on MRI. Besides, there is also focal tracer accumulation in the ulnar-sided base of the 2^nd^ metacarpal (d) indicating a “carpal boss”, which was not in the main clinical focus at that time.

The correct localization of lesions (spatial accuracy) was highest in SPECT/CT, slightly outperforming MRI. Especially lesions located in the ulnocarpal compartment (Figures **1** and **2**) and DRUJ were detected correctly by experienced readers to a greater extent than by inexperienced readers, both with SPECT/CT (mean value 75% vs. 44%) and MRI (mean value 69% vs. 44%). The situation was different concerning the carpal bones: Lesions in the proximal row were properly assessed only by experienced readers on MRI (mean value 70%; SPECT/CT: 40%), in the distal row only by SPECT/CT (mean value 80%; MRI: 60%). Details are given in table **3**. 

**Table 3:** Geographic distribution of relevant lesions according to the standard of reference, mean detection rate by all modalities for experienced and inexperienced readers.

**Location of relevant lesion**	**Number of relevant lesions / standard of reference**	**Percentage of relevant lesions detected (mean percent values based on two readers in each group)**										
		**Plain radiographs**		**Bone scan**		**CT**			**SPECT/CT**		**MRI**	
		Experienced readers	Inexperienced readers	Experienced readers	Inexperienced readers	Experienced readers		Inexperienced readers	Experienced readers	Inexperienced readers	Experienced readers	Inexperienced readers
Ulnocarpal compartment + DRUJ	8	31	38	44	31	44		31	75	44	69	44
Distal ulnar groove	1	0	0	0	0	0		0	0	0	100	0
Radiocarpal compartment	3	50	50	100	50	67		67	100	63	50	50
Proximal carpal row	5	20	0	20	20	20		10	40	40	70	40
Distal carpal row	5	0	0	20	30	0		0	80	30	60	20
Metacarpophalangeal	4	75	50	75	63	75		38	100	63	75	25
Generalized	1	0	0	100	50	0		0	100	0	0	0
		**Mean detection rate ± SD**										
**All lesions**	**27 (100%)**	**30% ± 5%**	**24% ± 3%**	**46% ± 3%**	**35% ± 3%**	**35% ± 3%**	**24% ± 8%**		**74% ± 0%**	**44% ± 10%**	**65% ± 3%**	**33% ± 26%**
		**p = 0.26**		**p = 0.06**		**p = 0.08**			**p = 0.004**		**p = 0.001**	

Results are given as mean per-cent values. DRUJ: distal radioulnar joint, SD: standard deviation.

Concerning the evaluation of the type of the lesions, there was no significant difference found between SPECT/CT and MRI in general. SPECT/CT performed better in the evaluation of predominantly bony lesions, while MRI showed its known strength in soft tissue pathologies. 

MRI was slightly superior to SPECT/CT concerning the evaluation of the assumed etiology of lesions, when read by experienced individuals (correct etiology in 39 of 2x 27 lesions (72%), and 37 of 2x 27 lesions (69%)). Generally, post-traumatic lesions were evaluated with a higher accuracy on SPECT/CT than on MRI, while degenerative lesions showed better results upon evaluation with MRI compared to SPECT/CT. 

### Interobserver agreement

**Figure 3:**
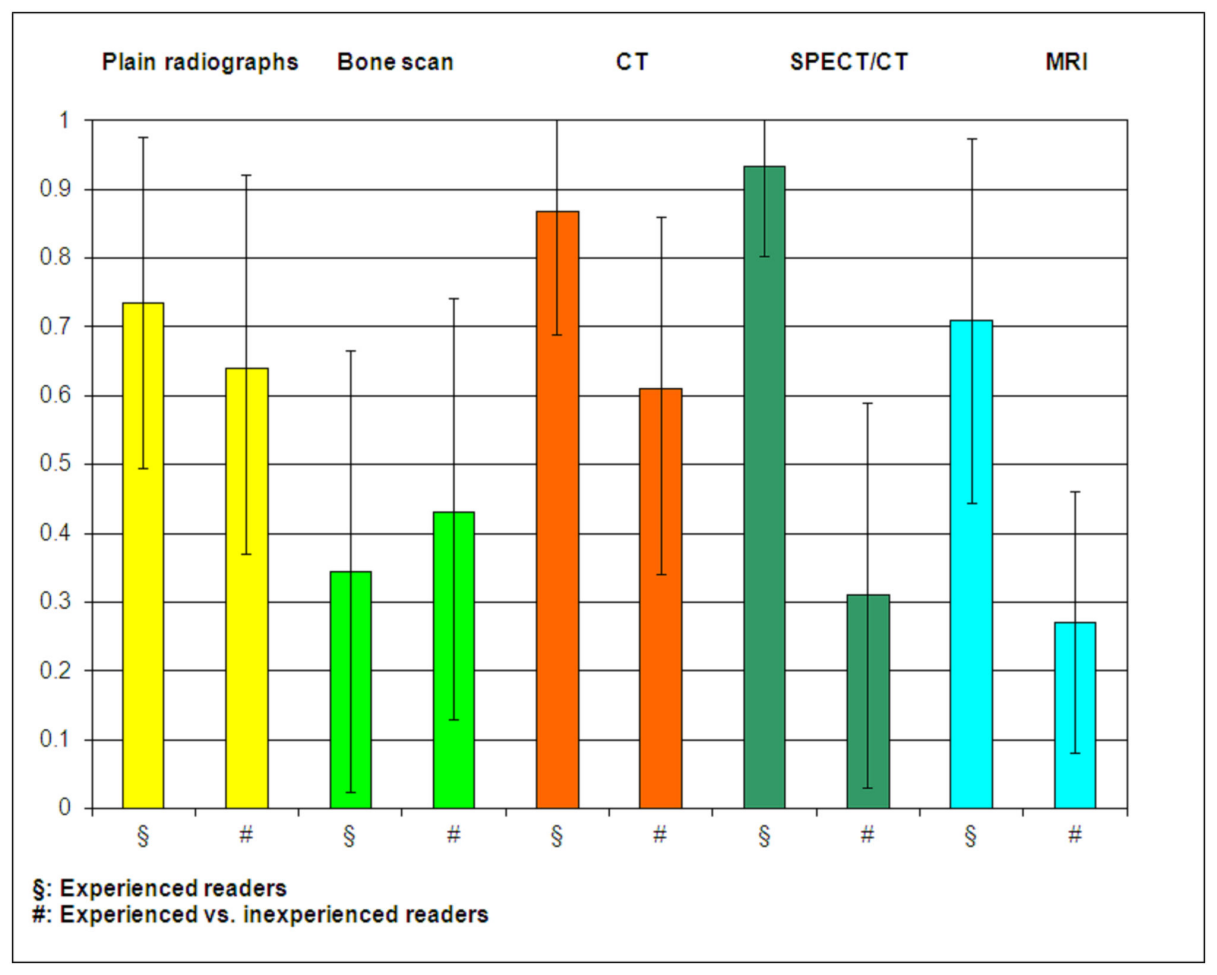
Kappa of interobserver agreement in detection of the relevant lesion. The error bars delineate the 95% confidence interval.

Detailed results are given in table **4**. Kappa for interobserver agreement in lesion detection are depicted in Figure **3**. 

**Table 4:** Kappa of agreement between experienced readers, and between experienced and inexperienced readers.

	**Plain radiographs**		**Bone scan**		**CT**		**SPECT/CT**		**MRI**	
	Experienced readers	Experienced vs. inexperienced readers	Experienced readers	Experienced vs. inexperienced readers	Experienced readers	Experienced vs. inexperienced readers	Experienced readers	Experienced vs. inexperienced readers	Experienced readers	Experienced vs. inexperienced readers
	Kappa (95% CI)	Mean kappa	Kappa (95% CI)	Mean kappa	Kappa (95% CI)	Mean kappa	Kappa (95% CI)	Mean kappa	Kappa (95% CI)	Mean kappa
**Detection of lesion**	0.73 (0.49 - 0.98)	0.64	0.34 (0.02 - 0.67)	0.43	0.87 (0.69 - 1.00)	0.61	0.93 (0.80 - 1.00)	0.31	0.71 (0.44 - 0.97)	0.27
**Location of lesion**	0.69 (0.42 - 0.97)	0.62	0.63 (0.36 - 0.89)	0.50	0.87 (0.69 - 1.00)	0.61	0.91 (0.75 - 1.0)	0.36	0.75 (0.52 - 0.98)	0.26
**Type of lesion**	0.42 (0.09 - 0.76)	0.26	0.51 (0.22 - 0.80)	0.59	0.74 (0.50 - 0.98)	0.53	0.69 (0.41 - 0.97)	0.40	0.75 (0.52 - 0.98)	0.15
**Etiology of lesion**	0.47 (0.15 - 0.78)	0.42	0.50 (0.20 - 0.80)	0.57	0.54 (0.24 - 0.84)	0.67	0.85 (0.64 - 1.00)	0.27	0.74 (0.51 - 0.97)	-0.01

CI: confidence interval.

#### Plain radiographs

There was similar substantial agreement on detection of the relevant lesion and its location between experienced observers (kappa 0.69, 0.73, respectively), and between experienced and inexperienced observers (mean kappa 0.64, 0.62, respectively). The agreement on type and etiology of lesions was generally poor between experienced readers, as well as between experienced and inexperienced readers. 

#### Bone scan

Bone scan yielded substantial agreement between the experienced readers on location (0.63), and moderate agreement on type (0.51) and on etiology (0.50). The agreement between both observer groups was moderate on all criteria. 

#### CT

While the diagnostic accuracy was insufficient, there was almost perfect agreement between the experienced readers on lesion detection and location (0.87), substantial agreement on lesion type (0.74), and moderate agreement on lesion etiology (0.54). Between both reader groups, there was substantial agreement on all criteria except lesion type. 

#### SPECT/CT

In contrast to the aforementioned modalities, the accuracy of experienced readers was relatively high in SPECT/CT (lesion detection 0.93, localisation 0.91, etiology 0.85). Interestingly, no agreement on any of the criteria was found between the two reader groups. 

#### MRI

While MRI was the modality with the second best - though merely low - accuracy of experienced readers, there was substantial agreement between the experienced readers on all criteria (0.71 - 0.75). In contrast, there was no valuable agreement between experienced and inexperienced readers. 

## Discussion

### Diagnostic accuracy

The crucial point in the imaging of wrist pain is to allocate one or more causative or active lesions to a patient’s symptoms. Establishing the diagnosis in morphological imaging can be difficult, especially in patients with chronic pain, because morphological changes are known to lag behind metabolic activity. In patients with low back pain due to neoplastic lesions, SPECT has been shown to have similar results as MRI [10,11]. 

Bone scans using Tc-99^m^-DPD are ideal for detecting active bone turnover. CT alone is limited in musculoskeletal conditions due to its low soft tissue contrast. Thus, isolated cartilage and ligament lesions are not easily recognized on CT without intra-articular contrast medium. In patients with acute wrist trauma and persisting pain, studies have shown that CT is superior to bone scan in diagnosing occult fractures, and slightly inferior to MRI [12,13]. However, CT is considerably less sensitive than MRI in detecting cancellous bone lesions such as trabecular fractures [14,15]. This limitation may be overcome by hybrid imaging obtained with a SPECT/CT system, combining metabolic information with precise anatomical location of tracer uptake.

As shown in another recent study [6], SPECT/CT was the most specific modality in such a selected patient population (specificity 90% in the present study), with a very high PPV of 0.98, whereas MRI was highly non-specific (10%) for experienced readers. In contrast, the specificity of MRI for inexperienced readers (80%) was found slightly higher than that of SPECT/CT (60%). We conclude that if an inexperienced reader discovers a pathology on MRI, it will likely correspond to the clinically leading pathology. This especially holds true when considering that the overall mean detection rates for experienced and inexperienced readers were not significantly different (44% vs. 38%). However, the low specificity of MRI in the experienced group reveals one dilemma of this modality: through its ability to show a lot of different pathologies in different compartments, it becomes hard to grade the severity of all those pathologies and then pinpoint the clinically relevant one in patients with several co-existing lesions. The human body provides many "false positives" secondary to the aging process and remote trauma. CT scans and bone scans inherently will provide a more binary result given their more limited ability to detect pathology compared with MRI. This allows for a more limited range of interpretations, contrary to MRI. 

SPECT/CT and MRI were the only modalities with reasonable sensitivity, with SPECT/CT slightly outperforming MRI (sensitivity: 74% vs. 65%, respectively; accuracy: 77% vs. 56%, respectively). The registration technique in hybrid SPECT/CT already proved to be more accurate than methods used previously [16–19]. Utsunomiya and co-workers found fused SPECT and CT images especially useful for the differentiation of osteoarthritis from malignant conditions [20]. A recent study by Linke et al. also demonstrated that SPECT/CT increases the diagnostic accuracy in orthopaedic disorders of the extremities [21]. The authors reported a revision of the diagnostic category in one third of patients. These findings parallel our results. False negatives in MRI in our study were particularly lesions with bone remodelling and many similarly appearing degenerative or posttraumatic changes in one or more compartments, but with distinct tracer uptake of one of these lesions in SPECT/CT. Consequently, SPECT/CT was superior to MRI in bone-only lesions and in bone lesions combined with other types of pathology. The overall etiology of lesions was correctly identified by SPECT/CT and MRI to a similar extent, but with advantages for MRI. MRI may depict a multitude of soft tissue pathologies not detectable by CT and / or bone scan. Thus, MRI provides a much broader view than only bone changes, and can give pertinent clinical information not detectable by a CT scan or a bone scan. This can however be foiled by several co-existing lesions in the same patient, as mentioned above. Degenerative lesions in the wrist usually originate at chondral or ligamentous surfaces and involve osseous structures in the later course. The latter may be an explanation for the limited usefulness of SPECT/CT in these lesions in our patient group. 

### Interobserver agreement

 The lack of exact anatomic localization of a lesion has been a major disadvantage of bone scans concerning the interobserver agreement [18,20,22]. This is due to the fact that a hot spot at one place, e.g. the lunate bone, could result from different pathologies (e.g. Kienböck’s disease, osteoarthritis, fracture etc.). This limits the interpretation in the absence of sufficient anatomical and morphological information [23]. We focussed on the agreement between experienced readers, as well as on the agreement between experienced and inexperienced readers to determine if the lack of training may be overcome by combined metabolic and morphological information. Fused SPECT and CT images were already shown to increase the interobserver agreement and observer confidence in suspected neoplastic bone lesions, compared to separate sets of scintigraphic and CT images [20]. SPECT/CT was also demonstrated to have a higher interobserver agreement and intraobserver agreement than bone scan, CT and a combination of both in disorders of the foot and ankle [7]. However, MRI was not assessed in that study. 

In the present study we also found that SPECT/CT is the modality with the highest agreement between experienced readers. The second highest agreement was found in CT, however, with lower accuracy. A higher interobserver agreement with values of 1.00 for TFCC and cartilage lesions and 0.89 for ligament lesions was found in the study by De Filippo and co-workers analyzing CT arthrography in patients with degenerative or posttraumatic arthropathy of the wrist [24]. These differences are probably based on the use of contrast medium. The inherent limitations of naïve CT may be overcome by the addition of functional information in SPECT/CT, which in turn increases both interobserver agreement and accuracy. This holds obviously true only for readers with a certain level of experience since we could not demonstrate a fair agreement on any of the analyzed criteria between readers with different levels of experience, both in SPECT/CT and MRI. However, this may not be the case when assessing only patients with degenerative lesions [7]. 

Only for the typification of lesions, MRI showed a hardly higher interobserver agreement for experienced readers (0.75) than SPECT/CT (0.69). However, the agreement between the experienced and inexperienced readers was again higher in SPECT/CT (for all evaluated criteria), pointing out that SPECT/CT might be at least slightly easier to understand and to read than MRI. However, in another study assessing cartilage lesions of the wrist, MRI had a high specificity, but low interobserver agreement [25]. A fair interobserver agreement was found for synovial lesions of the hand [26]. Finding an “active” osseous lesion in MRI is predominantly based on the prevalence of “bone marrow edema”, a pattern of ill-defined hyperintense signal on T2-weighted images. However, this feature is considered non-specific [27–31]. Bone marrow edema and radiotracer uptake do not necessarily prevail concurrently [6]. Supposedly this partially explains the observed relatively high interobserver agreement but low specificity of MRI. However, it is well established that bone scan and MRI findings may have little clinical relevance, particularly in the absence of pertinent history. 

### Limitations

Besides the relatively small number of patients, the study was performed retrospectively. The results of all imaging modalities are partly an inherent bias on the standard of reference of this study, i. e. on the clinical course. However, this is a general limitation of retrospective diagnostic imaging studies. Another limitation may be the time gap between the examinations with median intervals of 32 to 37 days. Lesions certainly may change during this period. However, all patients had chronic, clinically relevant symptoms at any time point of imaging. Furthermore, the scarcity of inflammatory lesions in our cohort tends to bias the results away from MRI as a useful modality. 

## Conclusion

Overall, SPECT/CT yielded the highest diagnostic accuracy in both experienced and inexperienced readers. The method was found reliable, providing higher interobserver agreement than all other imaging modalities, being outperformed only by MRI concerning the typification of lesions. However, training and experience are mandatory for the correct reading of SPECT/CT. SPECT/CT may be integrated into the diagnostic imaging algorithm of patients with chronic wrist pain, especially if MRI results are equivocal.
